# Machine learning pipeline for blood culture outcome prediction using Sysmex XN-2000 blood sample results in Western Australia

**DOI:** 10.1186/s12879-023-08535-y

**Published:** 2023-08-24

**Authors:** Benjamin R. McFadden, Timothy J. J. Inglis, Mark Reynolds

**Affiliations:** 1 School of Physics, Mathematics and Computing, University of Western Australia, Perth, Australia; 2 Western Australian Country Health Service, Perth, Australia; 3 School of Medicine, University of Western Australia, Perth, Australia; 4 Department of Microbiology, Pathwest Laboratory Medicine, Perth, Australia

**Keywords:** Blood cultures, Machine learning, Bloodstream infections, Diagnostic stewardship

## Abstract

**Background:**

Bloodstream infections (BSIs) are a significant burden on the global population and represent a key area of focus in the hospital environment. Blood culture (BC) testing is the standard diagnostic test utilised to confirm the presence of a BSI. However, current BC testing practices result in low positive yields and overuse of the diagnostic test. Diagnostic stewardship research regarding BC testing is increasing, and becoming more important to reduce unnecessary resource expenditure and antimicrobial use, especially as antimicrobial resistance continues to rise. This study aims to establish a machine learning (ML) pipeline for BC outcome prediction using data obtained from routinely analysed blood samples, including complete blood count (CBC), white blood cell differential (DIFF), and cell population data (CPD) produced by Sysmex XN-2000 analysers.

**Methods:**

ML models were trained using retrospective data produced between 2018 and 2019, from patients at Sir Charles Gairdner hospital, Nedlands, Western Australia, and processed at Pathwest Laboratory Medicine, Nedlands. Trained ML models were evaluated using stratified 10-fold cross validation.

**Results:**

Two ML models, an XGBoost model using CBC/DIFF/CPD features with boruta feature selection (BFS) , and a random forest model trained using CBC/DIFF features with BFS were selected for further validation after obtaining AUC scores of $0.76 \pm 0.04$ and $0.75 \pm 0.04$ respectively using stratified 10-fold cross validation. The XGBoost model obtained an AUC score of 0.76 on a internal validation set. The random forest model obtained AUC scores of 0.82 and 0.76 on internal and external validation datasets respectively.

**Conclusions:**

We have demonstrated the utility of using an ML pipeline combined with CBC/DIFF, and CBC/DIFF/CPD feature spaces for BC outcome prediction. This builds on the growing body of research in the area of BC outcome prediction, and provides opportunity for further research.

**Supplementary Information:**

The online version contains supplementary material available at 10.1186/s12879-023-08535-y.

## Introduction

Bloodstream infections (BSIs) are becoming an increasingly significant burden on the global population. At the local level, BSIs have significant costs to healthcare systems and patients. This is represented by both the economic impact as a result of diagnosis and treatment, and the damage to patients as a result of a BSI. Untreated BSIs can lead to serious health consequences. Sepsis, which is currently defined as a life threatening organ dysfunction due to a dysregulated immune response to infection [1], is one potential result of a BSI. BSIs are the result of infections with pathogenic organisms including bacteria and fungi. The detection of a BSI requires blood culture (BC) testing to identify infections in the bloodstream. The test uses a blood sample from the patient, placed in a medium to promote growth of microorganisms. This is incubated in the laboratory and observed for growth. BC testing is considered the current “gold standard” for diagnosis of BSIs, however, BC testing is generally overused and results in low positive yields [2, 3]. This can lead to longer hospital stays, additional unnecessary patient tests, increased costs and resource expenditure, and the unnecessary application of antimicrobials [3–6]. This, in turn, contributes to the proliferation of antimicrobial resistance (AMR), an increasing burden on the global population with an estimated 1$\cdot$27 million (0$\cdot$911-1$\cdot$71) deaths directly attributable to drug resistance in 2019 [7]. Implementing diagnostic stewardship regarding BC tests has therefore become a significant clinical priority. The aim of diagnostic stewardship is to *“select the right test for the right patient, generating accurate, clinically relevant results at the right time to optimally influence clinical care and to conserve health care resources”* [8]. In the case of BC testing, it is important to identify when BC tests are unnecessary, in order to support clinicians deciding whether to order BCs [9]. With the increasing amount of data being produced and stored in the clinical laboratory environment, machine learning (ML) algorithms can be utilised for diagnostic stewardship of BSIs. ML solutions are increasingly applied for problems in infection science. In the hospital, ML models are used to assist in the patient diagnosis, treatment, and management; and in the clinical laboratory, ML is providing solutions for problems relating to laboratory workflows and testing methodologies. In particular, the analysis of large, multidimensional datasets that are difficult for humans to analyse provides the opportunity for ML based approaches. This paper introduces a ML pipeline for BC outcome prediction using blood sample data produced by Sysmex-XN 2000 hematology analysers (Sysmex, Kobe, Japan). The ML models within this pipeline have been trained on retrospective data, in addition to being validated on retrospectively collected, internal, and external datasets. The purpose of this pipeline is to reduce the number of unnecessary BCs, and improve diagnostic stewardship practices of BC testing.

## Method

### Machine learning lifecycle

We present a ML pipeline for BC outcome prediction which includes data processing, and model development and evaluation. Each of these components are discussed in following sections.

### Data collection and processing

We trained ML models using complete blood count (CBC), white blood cell differential (DIFF), and cell population data (CPD) produced by the Sysmex XN-2000 hematology analysers. CBC and DIFF features are routinely reported in the laboratory environment, while CPD features are not routinely reported, as they are currently only used for research purposes. Three separate datasets were utilised, including training, internal validation, and external validation datasets, all obtained retrospectively. Properties of these datasets are discussed in the following sections. The ML model development process is discussed in the section *Machine learning model development*. All data was produced between 1 January 2018 and 31 May 2020. CBC, DIFF, and CPD test results were joined with respective microbiological outcome data from the laboratory information system (LIS). Test results and corresponding BC outcomes were included if the blood samples for CBC and BC testing were taken at the same time, therefore sharing a sample identification number. Imputation of missing values was not required as all features that were included during the training phase were complete when tests were performed. Data used throughout this study was managed appropriately based on local research procedures and guidelines. All data was provided in a de-identified form, and additional demographic or clinical outcome data from patients was not used. These datasets have been previously utilised in unpublished research [10]. The datasets are described in the following section, and in Table 1. Only samples from adult populations (age > 18) were included, and samples were excluded if the CBC test did not have a corresponding BC test with matching sample identification. Samples were also excluded if errors were present during CBC data generation. These samples were automatically flagged by the analyser. We were unable to determine which organisms were clinically significant or contaminated. Therefore, based on a previous study by Nannan Panday et al. [11], we considered Micrococcus species, Bacillus species, Coagulase-negative staphylococci (CoNS), Corynebacterium species, and Propionibacterium acnes as non-significant/contamination. CBC data which had a corresponding BC result with these microorganisms were not considered in our study. This was done to reduce the risk of including incorrectly labelled data into the training dataset.

### Datasets

#### Retrospective training dataset

The retrospective training dataset includes results produced between 1 January 2018 and 31 December 2019. Data was generated at Pathwest Laboratory Medicine, Nedlands, Western Australia from patients at Sir Charles Gairdner Hospital (SCGH), a teaching hospital in Nedlands, Western Australia. The training set contains 10965 samples. 10134 of these blood samples were drawn with negative BC results (92.42%), and 831 were drawn with positive BC results (7.58%).

#### Retrospective internal validation dataset

The retrospective internal validation dataset includes results produced between 1 January 2020 and 31 May 2020. Data was generated at Pathwest Laboratory Medicine, Nedlands, Western Australia from patients at SCGH. This set contains 318 samples. 292 of these blood samples were drawn with negative BC results (91.82%), and 26 were drawn with positive BC results (8.18%).

#### Retrospective external validation dataset

The retrospective external validation dataset includes results produced between 1 January 2020 and 31 May 2020. Data was generated at Pathwest Laboratory Medicine centres in Western Australia outside of the Pathwest Laboratory Medicine, Nedlands centre. Data was extracted from the LIS. This set contains 1245 samples. 1138 of these blood samples were drawn with negative BC results (91.41%), and 107 were drawn with positive BC results (8.59%). For this dataset, a model trained on CBC and DIFF data was evaluated due to the inability to obtain CPD from other centres.

**Table 1:** Description and properties for each dataset

Dataset	Time period	Overview
Training	Between 1 January 2018 and 31 December 2019	The training set contains 10965 samples. 10134 of these blood samples were taken with negative BC results (92.42%), and 831 were drawn with positive BC results (7.58%).
Internal validation	Between 1 January 2020 and 31 May 2020	This set contains 318 samples. 292 of these blood samples were drawn with negative BC results (91.82%), and 26 were drawn with positive BC results (8.18%).
External validation	Between 1 January 2020 and 31 May 2020	This set contains 1245 samples. 1138 of these blood samples were drawn with negative BC results (91.41%), and 107 were drawn with positive BC results (8.59%).

### Interpretation of features

Hematology data produced by the Sysmex XN-2000 module analysers was used as the input for the ML models, including CBC, DIFF, and CPD features. A CBC is a regularly requested laboratory test that is used to analyse patient blood samples and reports information regarding the cells in the blood including white blood cells/leukocytes (WBC), platelets/thrombocytes (PLT), and red blood cells/erythrocytes (RBC). In addition to a standard CBC, a DIFF which provides information about the different WBC types is also often performed. This includes analysis of neutrophils (NEUT), lymphocytes (LYMPH), monocytes (MONO), basophils (BASO), and eosinophils (EO). From DIFF information, it is also possible to derive additional features including neutrophil-to-lymphocyte ratio (NLR), and monocyte-to-lymphocyte ratio (MLR). CPD features are produced as a result of the fluorescent flow cytometry method used by the Sysmex analysers. CPD provides numerical values for side scatter light (SSC), foward scatter light (FSC), and fluorescent light intensity (SFL) . These values are often presented graphically on a scattergram along the x-axis, z-axis, and y-axis respectively. SSC represents cellular granularity, FSC represents cell volume and shape, and SFL represents the nucleic acid and protein content of cells [12, 13]. Lastly, the Sysmex XN-2000 also generates interpretive program messages (IP flags) based on the outcome of a CBC analysis, and provides warnings for hematological conditions or disorders [14]. The analysers produce these flags for WBC, RBC, and PLT.

1 $$\begin{aligned} \text {Neutrophil-to-lymphocyte ratio (NLR)} = \frac{NEUT \, count}{LYMPH \, count} \end{aligned}$$

2 $$\begin{aligned} \text {Monocyte-to-lymphocyte ratio (MLR)} = \frac{MONO \, count}{LYMPH \, count} \end{aligned}$$

### Feature spaces

Two feature spaces were created and used to train ML models. The CBC and DIFF feature space (CBC/DIFF), and the CBC/DIFF feature space with the addition of CPD (CBC/DIFF/CPD). Separate models were trained on each of these feature spaces with a ML model development pipeline including feature selection and stratified 10-fold cross validation. The CBC and DIFF, and CPD features are shown in Tables 2 and 3 respectively. NLR and MLR are included as part of the CBC and DIFF features.

**Table 2:** Complete blood count (CBC) and differential (DIFF) features

Feature	Description
RDW-CV(%)	red blood cell distribution width
PLT$(10^9/L)$	Platelet count
MCHC(g/L)	mean corpuscular haemoglobin concentration
MCH(pg)	mean corpuscular haemoglobin
MCV(fL)	mean corpuscular volume
HGB(g/L)	haemoglobin
RBC$(10^{12}/L)$	Red blood cell count
WBC$(10^9/L)$	white blood cell count
MONO%(%)	monocyte differential relative percentage
BASO%(%)	basophil differential relative percentage
EO%(%)	Eosinophil differential relative percentage
LYMPH%(%)	lymphocyte differential relative percentage
NEUT%(%)	neutrophil differential relative percentage
BASO#$(10^9/L)$	absolute basophil count
MONO#$(10^9/L)$	absolute monocyte count
EO#$(10^9/L)$	absolute eosinophil count
LYMPH#$(10^9/L)$	absolute lymphocyte count
NEUT#$(10^9/L)$	absolute neutrophil count
NLR	neutrophil and lymphocyte ratio
MLR	monocyte and lymphocyte ratio

**Table 3:** Cell population data (CPD) features

Feature	Description
NE-SSC	Neutrophil complexity
NE-SFL	Neutrophil fluorescence intensity
NE-FSC	Neutrophils forward scatter
NE-WX	width of dispersion of neutrophil complexity
NE-WY	width of dispersion of neutrophils fluoresence
NE-WZ	width of dispersion of neutrophils size
LY-X	Lymphocytes complexity
LY-WX	Width of dispersion of lymphocytes complexity
LY-Y	Lymphocytes fluorescence intensity
LY-WY	Width of dispersion of lymphocytes fluorescence
LY-Z	Lymphocytes size
LY-WZ	Width of dispersion of lymphocytes size
MO-X	Monocytes complexity
MO-WX	Width of dispersion of monocytes complexity
MO-Y	Monocytes fluorescence intensity
MO-WY	Width of dispersion of monocytes fluorescence
MO-Z	Monocytes size
MO-WZ	Width of dispersion of Monocytes size

### Machine learning model development

Three different tree-based methods were evaluated; random forests (RF) [15], decision trees (DT) [16], and XGBoost (extreme gradient boosting) [17]. Only tree-based models were explored in this study as they provide the feature importance property after training the models. As the data is highly imbalanced, class weighting was implemented to manage this imbalance. The models were trained on each of the feature spaces, CBC/DIFF/CPD and CBC/DIFF. For each model and feature space, a feature selection method was selected. The methods include none (all features in the space included), recursive feature elimination (RFE) until 5 features, and the boruta feature selection method [18]. The boruta method was evaluated due to the effectiveness of the approach in previous studies in the medical domain [19–23]. The boruta method utilised RF and XGBoost models respectively when they were being trained. However, when training the DT models, RF was used with boruta to perform feature selection before training. This approach of using boruta with DT models has been previously implemented [24]. Stratified 10-fold cross validation of the training set was used to determine which models would be selected for further validation. The purpose of this study was to produce baseline ML models for BC outcome prediction. Given this objective, hyperparameter optimisation was not utilised due to the process being computationally expensive.

### Machine learning model evaluation

Models were evaluated using several metrics including area under the receiver operating characteristic curve (AUC), sensitivity, specificity, and the J-statistic. These metrics were calculated for stratified 10-fold cross validation during model training; and validation on the internal and external datasets. Metrics are for models when the classification threshold is at 0.5 unless otherwise stated.

### Software

The python programming language (version 3.10.5) was utilised for all software development in this study. Several python libraries were used including numpy (version 1.23.1) [25], pandas (version 1.4.3) [26, 27], scikit-learn (version 1.1.1) [28], XGBoost (version 1.6.1) [17], boruta_py (version 0.3), imbalanced-learn (version 0.9.1) [29], seaborn (version 0.11.2) [30], and matplotlib ( version 3.5.2) [31].

## Results

### Model training and cross validation

**Fig. 1:**
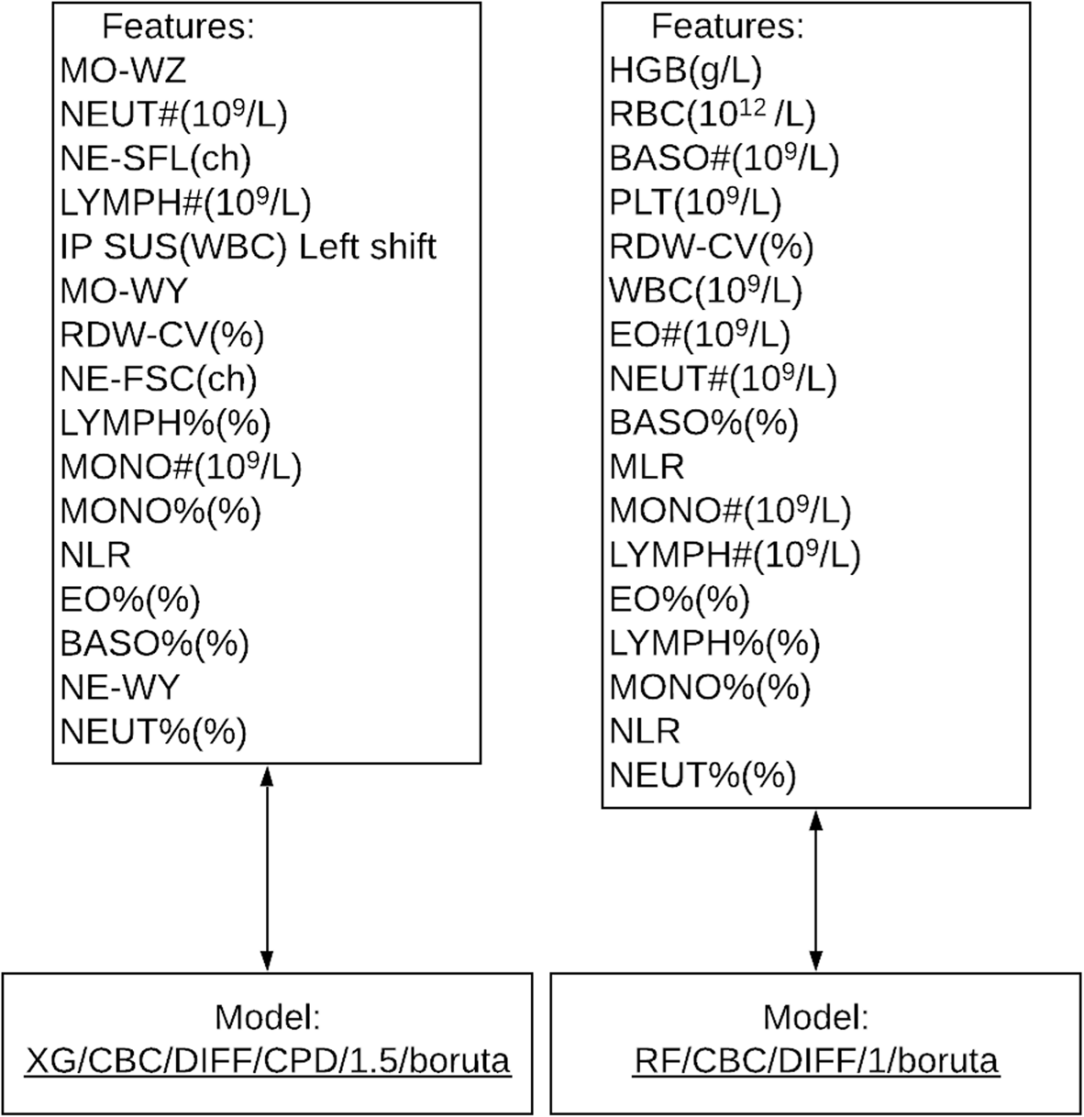
Flowchart demonstrating the features used in the XG/CBC/DIFF/CPD/1.5/boruta and RF/CBC/DIFF/1/boruta models

Results for the ML models after stratified 10-fold cross validation were sorted based on mean AUC, followed by the mean J statistic value, mean recall value, and mean diagnostic odds ratio at a classification threshold of 0.5. All of the ML models, feature selection methods, and class weight combinations performed similarly on stratified 10-fold cross validation. The lowest and highest AUC scores obtained were $0.70 \pm 0.05$ and $0.76 \pm 0.04$ respectively. Two models were subsequently selected for further evaluation. The first, which used the CBC/DIFF/CPD feature space was the XGBoost model with 1.5 class weights and utilising boruta for feature selection (XG/CBC/DIFF/CPD/1.5/boruta). This was selected as it was the best performing model when sorted accordingly. This represented a model which was balanced, with the possibility of adjusting thresholds for prediction. For external validation where CPD parameters were unavailable, the RF model with CBC and DIFF parameters was selected with balanced class weights and the boruta feature selection method (RF/CBC/DIFF/1/boruta). Table 4 shows the performance of these two models for stratified 10-fold cross validation during model training. Additional file 2 contains results for all models evaluated during the model training and cross validation stage. The features used in the XG/CBC/DIFF/CPD/1.5/boruta and RF/CBC/DIFF/1/boruta models are shown in Fig. 1. All feature importance’s for both models are shown in Tables 5 and 6.

**Table 4:** Performance of ML models for stratified 10-fold cross validation. Showing area under the receiver operating characteristic curve (AUC), J-statistic (J stat), sensitivity, and specificity at a classification threshold of 0.5

ML model	AUC	J stat at 0.5 threshold	Sensitivity at 0.5 threshold	Specificity at 0.5 threshold
XG/CBC/DIFF/CPD/1.5/boruta	$0.76 \pm 0.04$	$0.39 \pm 0.06$	$0.74 \pm 0.07$	$0.65 \pm 0.02$
RF/CBC/DIFF/1/boruta	$0.75 \pm 0.04$	$0.34 \pm 0.08$	$0.61 \pm 0.08$	$0.73 \pm 0.02$

**Table 5:** Feature importances for XG/CBC/DIFF/CPD/1.5/boruta. Features listed in table include width of dispersion of monocytes size ([MO-WZ]); absolute neutrophil count ($NEUT\#(10^9/L)$); neutrophil fluorescence intensity ([NE-SFL(ch)]); absolute lymphocyte count ($LYMPH\#(10^9/L)$); suspected presence of immature neutrophils (IP SUS(WBC)Left Shift); width of dispersion of monocyte fluorescence ([MO-WY]); red blood cell distribution width (RDW-CV(%)); neutrophils forward scatter ([NE-FSC(ch)]); relative percentage of lymphocytes (LYMPH%(%)); absolute monocyte count ($MONO\#(10^9/L)$); relative percentage of monocytes (MONO%(%)); neutrophil lymphocyte ratio (NLR); relative percentage of eosinophils(EO%(%)); relative percentage of basophils (BASO%(%)); width of dispersion of neutrophils fluorescence ([NE-WY]); relative percentage of neutrophils (NEUT%(%))

Feature	Importance
[MO-WZ]	0.036
$NEUT\#(10^9/L)$	0.037
[NE-SFL(ch)]	0.038
$LYMPH\#(10^9/L)$	0.038
IP SUS(WBC)Left Shift	0.041
[MO-WY]	0.044
RDW-CV(%)	0.044
[NE-FSC(ch)]	0.049
LYMPH%(%)	0.050
$MONO\#(10^9/L)$	0.056
MONO%(%)	0.065
NLR	0.067
EO%(%)	0.068
BASO%(%)	0.071
[NE-WY]	0.084
NEUT%(%)	0.213

**Table 6:** Feature importances for RF/CBC/DIFF/1/boruta. Features listed in table include hemoglobin (HGB(g/L)); red blood cell count ($RBC(10^{12}/L)$); absolute basophil count ($BASO\#(10^9/L)$); platelet count ($PLT(10^9/L)$); red blood cell distribution width (RDW-CV(%)); white blood cell count ($WBC(10^9/L)$); absolute eosinophil count ($EO\#(10^9/L)$); absolute neutrophil count ($NEUT\#(10^9/L)$); relative percentage of basophils (BASO%(%)); monocyte lymphocyte ratio (MLR); absolute monocyte count ($MONO\#(10^9/L)$); absolute lymphocyte count ($LYMPH\#(10^9/L)$); relative percentage of eosinophils (EO%(%)); relative percentage of lymphocytes (LYMPH%(%)); relative percentage of monocytes (MONO%(%)); neutrophil lymphocyte ratio (NLR); relative percentage neutrophils (NEUT%(%))

Feature	Importance
HGB(g/L)	0.003
$RBC(10^{12}/L)$	0.003
$BASO\#(10^9/L)$	0.008
$PLT(10^9/L)$	0.008
RDW-CV(%)	0.009
$WBC(10^9/L)$	0.015
$EO\#(10^9/L)$	0.026
$NEUT\#(10^9/L)$	0.028
BASO%(%)	0.030
MLR	0.036
$MONO\#(10^9/L)$	0.058
$LYMPH\#(10^9/L)$	0.063
EO%(%)	0.071
LYMPH%(%)	0.127
MONO%(%)	0.132
NLR	0.158
NEUT%(%)	0.224

### Model validation: internal dataset

**Fig. 2:**
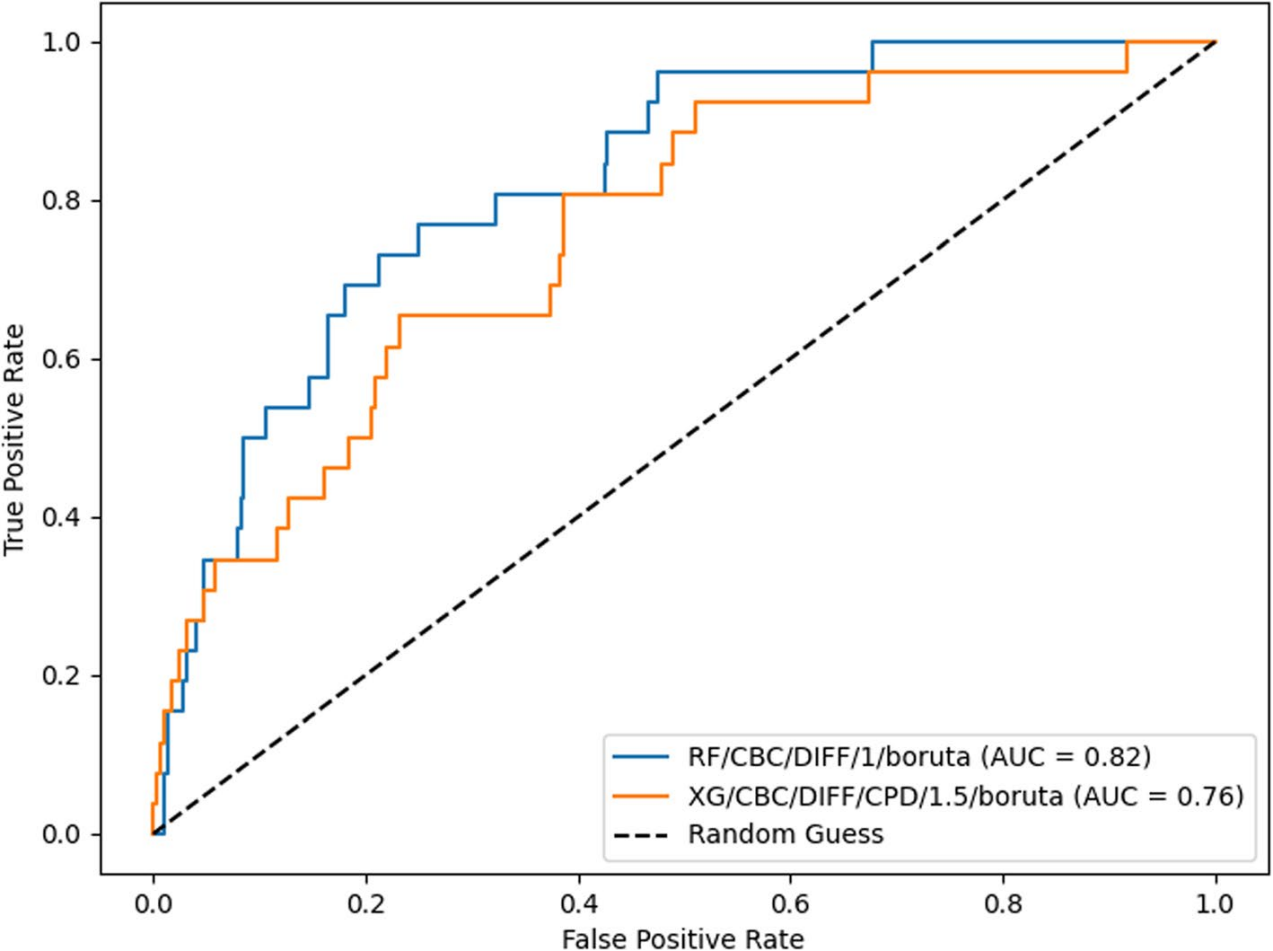
AUC curve for the XG/CBC/DIFF/CPD/1.5/boruta and RF/CBC/DIFF/1/boruta models when tested on the internal validation dataset. A positive prediction represents a positive blood culture outcome

The XG/CBC/DIFF/CPD/1.5/boruta and RF/CBC/DIFF/1/boruta models were evaluated on the internal validation set. The models achieved AUC scores of 0.76 and 0.82 respectively. AUC curves for these models are shown in Fig. 2. At the classification threshold of 0.5, the models achieved sensitivity scores of 0.81 and 0.77, and specificity scores of 0.61 and 0.69 respectively (Additional file 1, Figs. 1 and 2 for confusion matrices). At the classification threshold of 0.4, the models achieved sensitivity scores of 0.92 and 0.96, and specificity scores of 0.48 and 0.52 respectively (Additional file 1, Figs. 3 and 4 for confusion matrices). At the classification threshold of 0.3, the models achieved sensitivity scores of 0.96 and 1.0, and specificity scores of 0.31 and 0.15 respectively (Additional file 1, Figs. 5 and 6 for confusion matrices).

### Model validation: external dataset

**Fig. 3:**
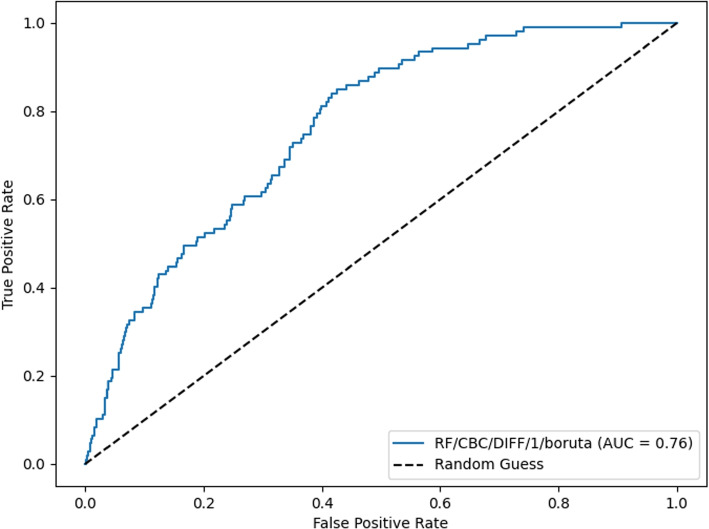
AUC curve for RF/CBC/DIFF/1/boruta model when tested on the external dataset. A positive prediction represents a positive blood culture outcome

The RF/CBC/DIFF/1/boruta model was evaluated on the external validation dataset as CPD parameters were unavailable. The model achieved an AUC score of 0.76. The AUC curve is shown in Fig. 3. At the classification threshold of 0.5, the model achieved sensitivity and specificity scores of 0.62, 0.70 respectively (Additional file 1, Fig. 7 for confusion matrix). At the classification threshold of 0.4, the model achieved sensitivity and specificity scores of 0.87 and 0.54 respectively (Additional file 1, Fig. 8 for confusion matrix). At the classification threshold of 0.3, the model achieved sensitivity and specificity scores of 0.99, 0.24 respectively (Additional file 1, Fig. 9 for confusion matrix).

## Discussion

**Fig. 4:**
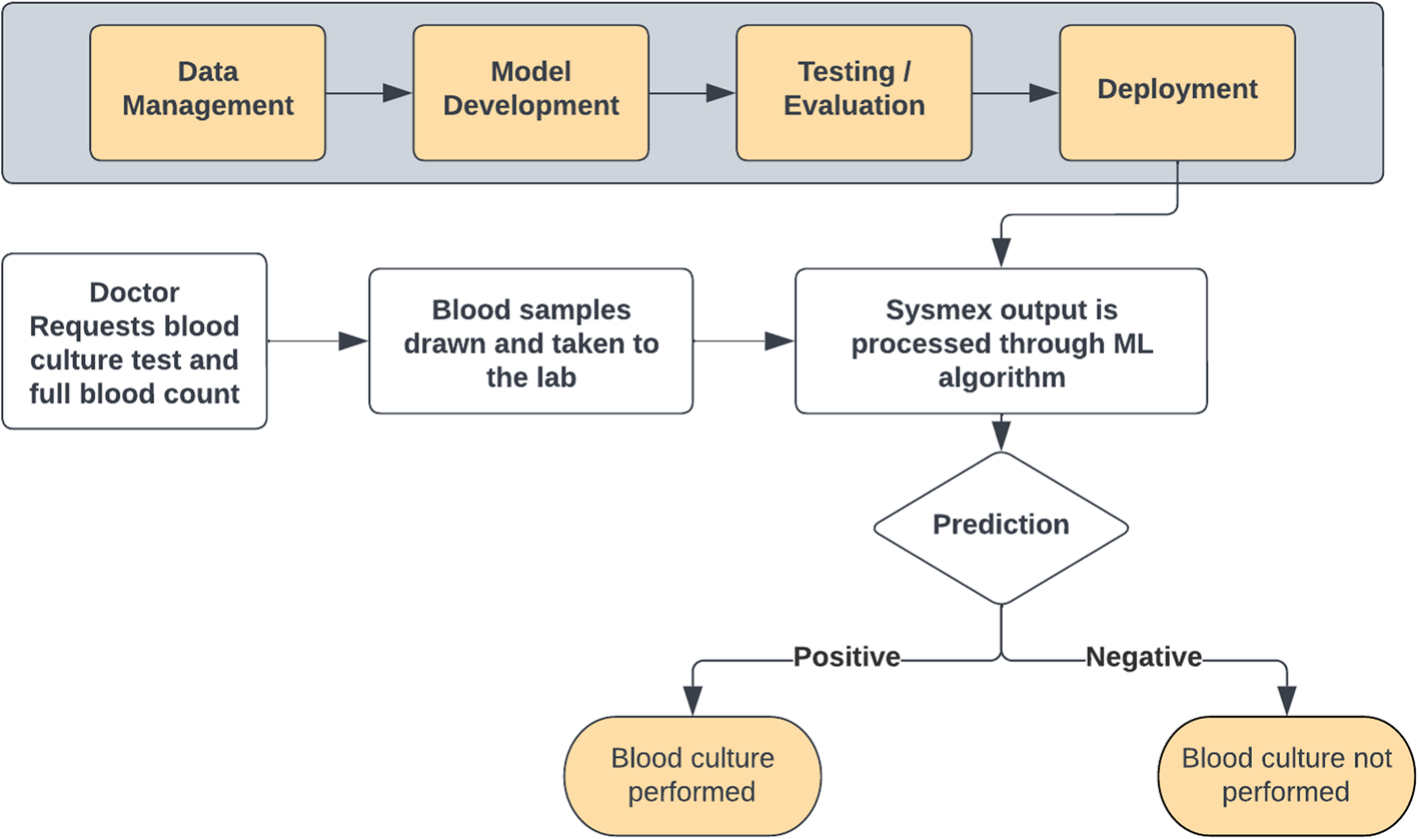
A potential clinical integration workflow for the proposed BC outcome prediction ML model

The ML pipeline established is this study performed consistently on stratified 10-fold cross validation, internal, and external validation datasets utilising CBC, DIFF, and CPD features produced by the Sysmex XN-2000 analysers. The pipeline is positioned to be validated in prospective studies for BC outcome prediction on patients who have BC and CBC samples drawn at the same time. This work adds to the existing body of literature, and presents, at the time of writing, the first use of CBC, DIFF, and CPD with ML for BC outcome prediction for the purpose of reducing the number of unnecessary BC tests. These results highlight the use of this approach for improvements in diagnostic stewardship by reducing the number of unnecessary BCs that are processed after BC tests have been requested by clinicians. All trained models demonstrated similar performance across all of the datasets. The XG/CBC/DIFF/CPD/1.5/boruta achieved an AUC score of 0.76 ± 0.04 on stratified 10-fold cross validation, and an AUC score of 0.76 on the internal test set. The RF/CBC/DIFF/1/boruta model obtained an AUC score of 0.75 ± 0.04 on 10-fold cross validation, and AUC scores of 0.82 and 0.76 on the internal and external datasets respectively. The feature importance scores for the two models supports previous findings in the literature. NEUT%(%) and NE-WY, both the first and second most important features for the XG/CBC/DIFF/CPD/1.5/boruta model, have been identified as important features in the identification of BSI [32], in addition to the other CPD features, which have shown effectiveness for the identification of BSI, sepsis, and most recently, SARS-CoV-2 [12, 32–34]. NLR, which has been previously identified as useful for the identification of BSI in patients with fever, was the second most important feature for the RF/CBC/DIFF/1/boruta model behind NEUT%(%) [35]. The application of ML for BC outcome prediction and identification of BSIs has also increased in recent years. Lien et al. [36] utilised ML for bacteremia detection utlising CBC and DIFF data but did not include NLR, MLR, or CPD. Boerman et al. [37] developed ML models for BC outcome prediction, where the patient population had already had BC tests requested by clinicians . The authors used hematological, biochemical, and physiological features to produce gradient boosted trees, and logistic regression models which obtained AUC scores of 0.77 and 0.78 respectively on test sets. Lastly, Schinkel et al. [38] developed an XGBoost model that obtained AUC scores of 0.81, 0.80, and 0.76 across testing, external, and prospective datasets, leading to a potential reduction of unnecessary BC tests by at least 30%. Typically, patient history, performing a physical assessment, and evaluating the results of laboratory tests are all considered when determining if and when a BC test should be performed [39]. In the proposed pipeline, only the results of routine blood tests are considered. A benefit of using only hematological data is that it simplifies the clinical integration process as the ML models do not rely on the production of data from multiple sources. Using a single source of data provides a simplified workflow for analysis and subsequent reduction in difficulty to integrate the approach within clinical laboratory workflows. Therefore, other features such as physiological, and biochemical features have been purposefully excluded from this study. A proposed clinical integration workflow is shown in Fig. 4, positioned between the physician and the laboratory, after blood tests have been performed.

Restricting the pipeline from using other, non-routinely collected data means that the proposed ML workflow from training, testing, and deployment, can be introduced more broadly as demonstrated by the performance of the pipeline on externally collected data. This study has limitations. Firstly, we utilised data produced from the Sysmex XN-2000 modules and did not take into consideration other information regarding the patient. We also focused on the entire hospital population. ML models may perform better when trained exclusively for certain patient sub populations. We have limited this study to focusing on data processing, model development, and model evaluation. Therefore we have not included discussion on methods of interpretability and explainabilty, and leave this open for future research. Deployment and integration strategies were not investigated and should be the focus of future work, along with evaluation of the ML pipeline in prospective studies. Furthermore, alternative feature selection methods, hyperparameter optimisation, and additional ML methods should be explored. Lastly, future work should aim to address the limitations surrounding the identification of clinically significant microorganisms and use a different method than the literature based approach we have chosen in this study.

## Conclusion

We have demonstrated the utility of ML approaches for BC outcome prediction, using routinely available hematology results produced by commonly used Sysmex XN-2000 analysers. Two ML models, one trained using CBC and DIFF features, and a model trained using CBC, DIFF, and CPD features demonstrated promising results. The ML pipeline established in this study provides a foundation for future clinical integration in the laboratory environment. Follow up research will evaluate this ML pipeline on a prospectively collected dataset. Future work will aim to further validate the findings presented in this paper and evaluate how the method could be implemented in practice. Particularly, it is important to determine if the method can be used safely and reliably to improve diagnostic stewardship regarding BC use and reduce the number of unnecessary BC tests.

### Supplementary information

**Additional file 1.** Confusion matrices for the XG/CBC/DIFF/CPD/1.5/boruta and RF/CBC/DIFF/1/boruta model predictions on the data presented.

**Additional file 2.** Contains results for all models evaluated during the model training and stratified 10-fold cross validation stage.

## Data Availability

Data that supports the findings outlined in this study has been produced by Pathwest Laboratory Medicine, and stored appropriately in Pathwest Laboratory Medicine facilities. Restrictions apply to the availability of the data used in this study. Data is therefore not publicly available, however, data may be made available upon request to the corresponding author, BRM, and subject to approval from Pathwest Laboratory Medicine. Requests for code can also be made to BRM.

## Abbreviations

AMR: Antimicrobial resistance
AUC: Area under the receiver operating characteristic curve
BASO: Basophils
BSI: Bloodstream infections
BC: Blood culture
CBC: Complete blood count
CBC/DIFF: CBC and DIFF feature space
CBC/DIFF/CPD: CBC, DIFF, and CPD feature space
CPD: Cell population data
DIFF: White blood cell differential
DT: Decision tree
EO: Eosinophils
FSC: Forward scatter light
IP flags: Interpretive program messages
LIS: Laboratory information system
LYMPH: Lymphocytes
ML: Machine Learning
MLR: Monocyte-to-lymphocyte ratio
MONO: Monocytes
NEUT: Neutrophils
NEUT%(%): Neutrophil percentage
NE-WY: Width of dispersion of neutrophils fluorescence
NLR: Neutrophil-to-lymphocyte ratio
PLT: Platelets/thrombocytes
RBC: Red blood cells/erythrocytes
RF: Random forest
RF/CBC/DIFF/1/boruta: Random forest model with balanced class weights, boruta feature selection, and CBC/DIFF feature space
RFE: Recursive feature elimination
ROC: Receiver operating characteristic
SCGH: Sir Charles Gairdner Hospital
SFL: Fluorescent light intensity
SSC: Side scatter light
WBC: White blood cell cells/leukocytes
XGBoost: Extreme gradient boosting
XG/CBC/DIFF/CPD/1.5/boruta: XGBoost model with 1.5 class weights, boruta feature selection, and CBC/DIFF/CPD feature space
