# Supplementary material for: Machine learning pipeline for blood culture outcome prediction using Sysmex XN-2000 blood sample results in Western Australia
Source: BMC Infect Dis. 2023 Aug 24;23:552. doi: 10.1186/s12879-023-08535-y (PMC10463910; doi:10.1186/s12879-023-08535-y)
Supplement: Supplementary file 1 — Additional file 1. Confusion matrices for the XG/CBC/DIFF/CPD/1.5/boruta and RF/CBC/DIFF/1/boruta model predictions on the data presented. [file 12879_2023_8535_MOESM1_ESM.docx]

Additional file 1:

Figure 1:


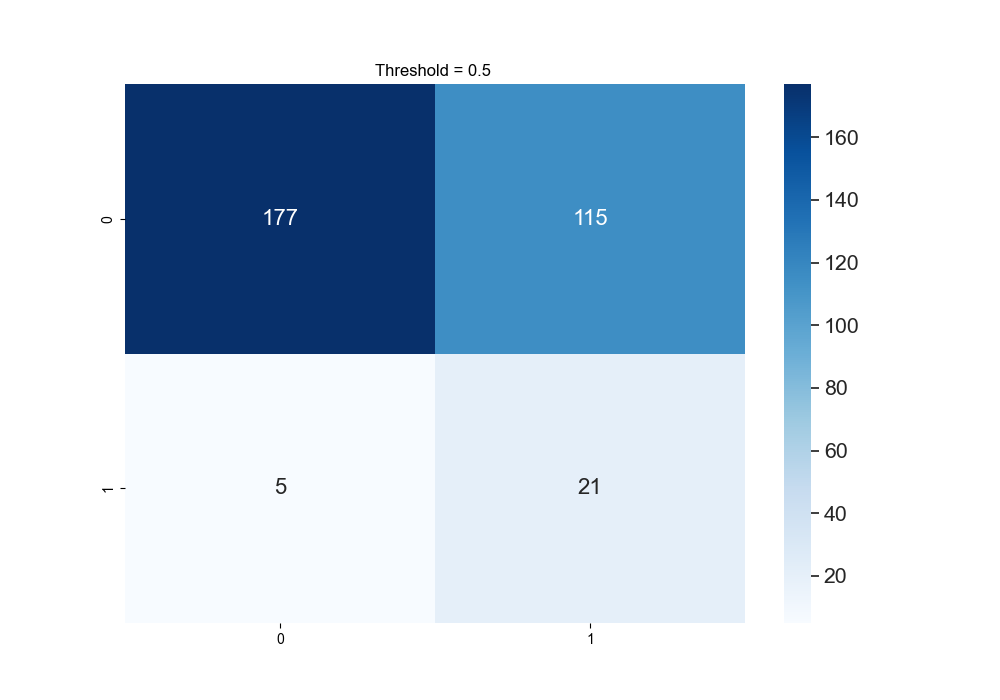


Confusion matrix for XG/CBC/DIFF/CPD/1.5/boruta model when tested on the external dataset at classification threshold of 0.5. Positive label 1 refers to a positive blood culture
result. Y axis represents actual value, X axis represents predicted value.

Figure 2:


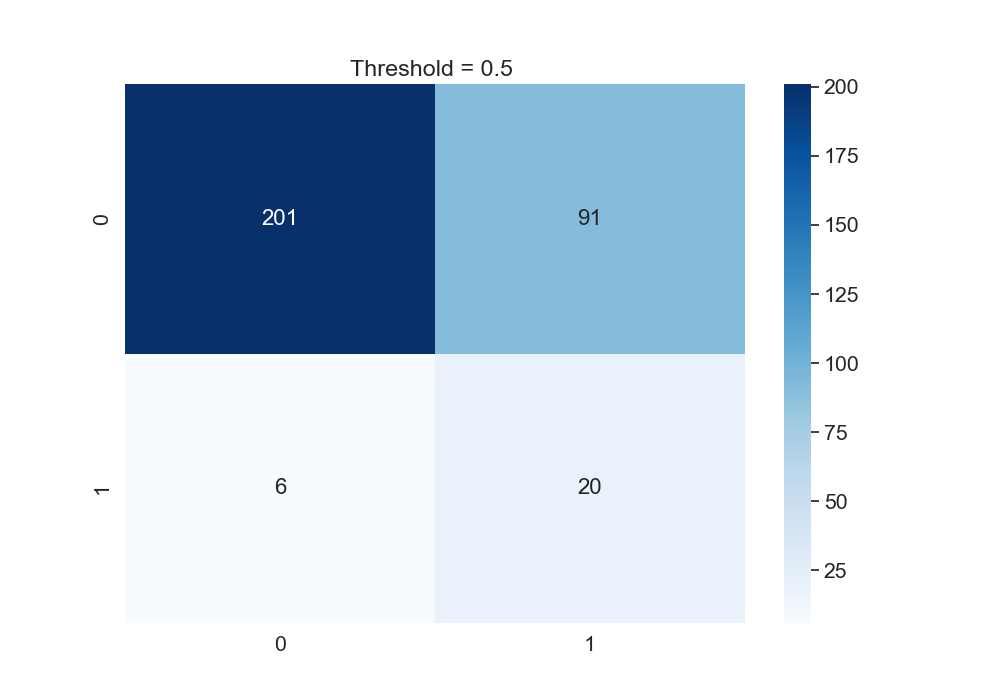


Confusion matrix for RF/CBC/DIFF/1/boruta model when tested on the external dataset at classification threshold of 0.5. Positive label 1 refers to a positive blood culture result. Y axis represents actual value, X axis represents predicted value.

Figure 3:


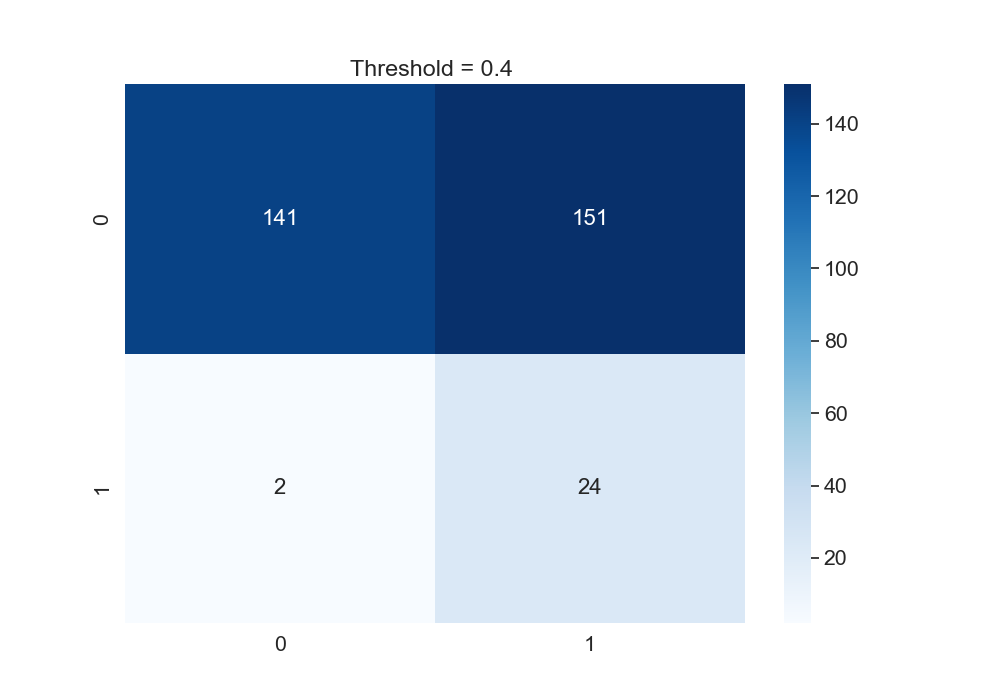


Confusion matrix for XG/CBC/DIFF/CPD/1.5/boruta model when tested on the external dataset at classification threshold of 0.4. Positive label 1 refers to a positive blood culture result. Y axis represents actual value, X axis represents predicted value.

Figure 4:


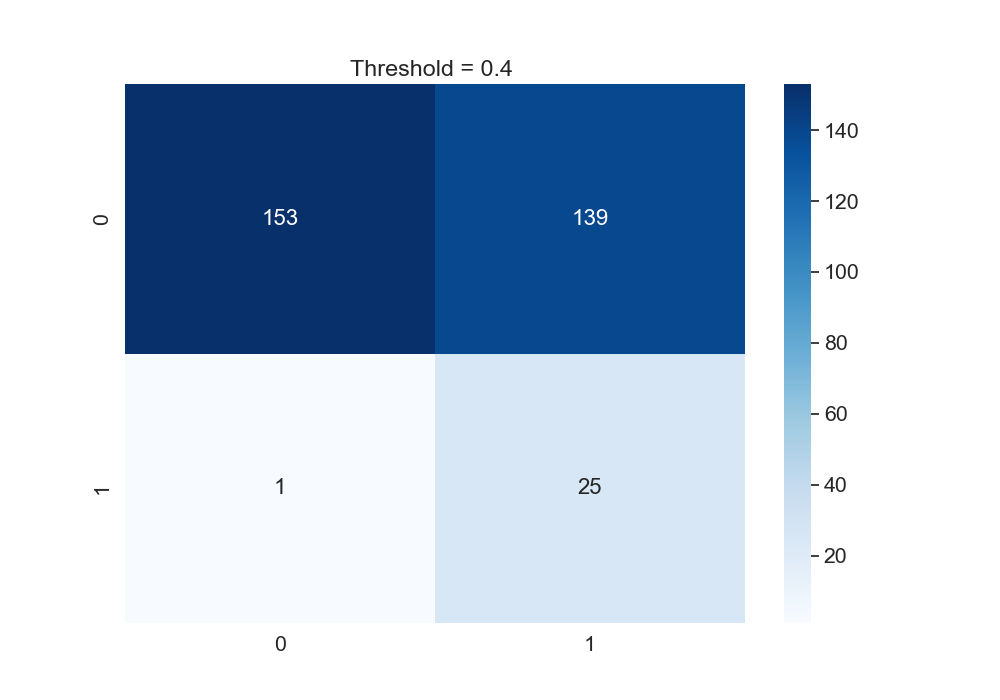


Confusion matrix for RF/CBC/DIFF/1/boruta model when tested on the external dataset at classification threshold of 0.4. Positive label 1 refers to a positive blood culture result. Y axis represents actual value, X axis represents predicted value.

Figure 5:


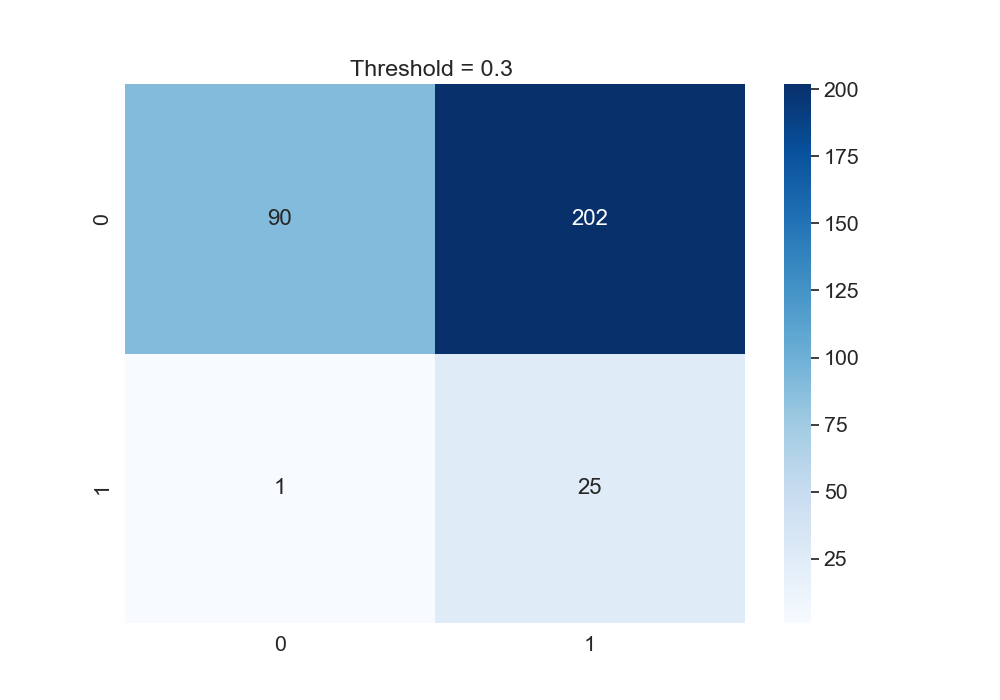


Confusion matrix for XG/CBC/DIFF/CPD/1.5/boruta model when tested on the external dataset at classification threshold of 0.3. Positive label 1 refers to a positive blood culture result. Y axis represents actual value, X axis represents predicted value.

Figure 6:


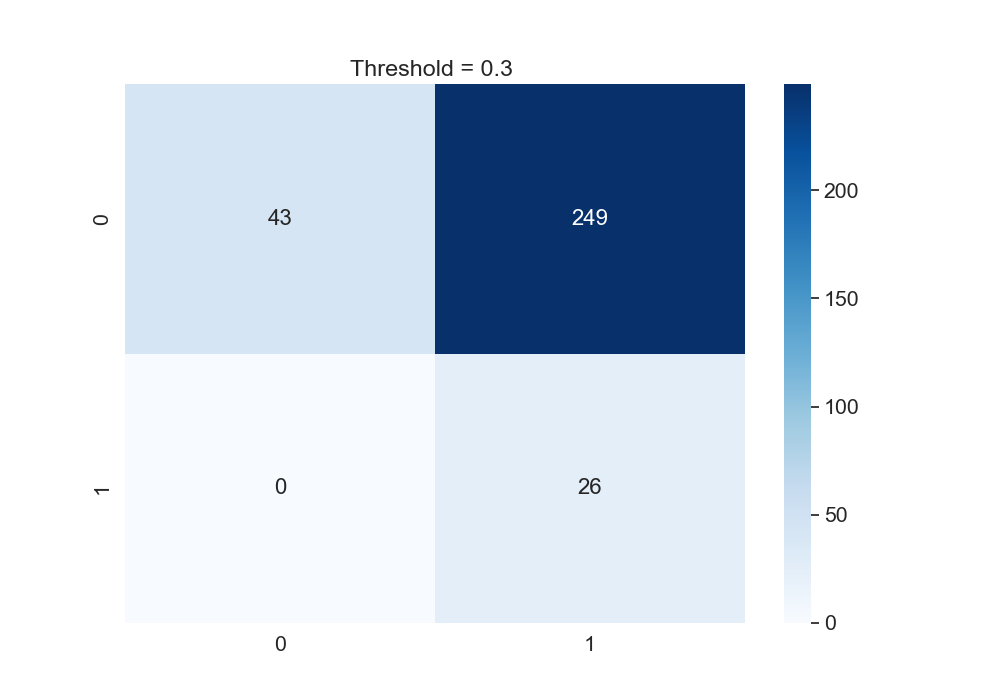


Confusion matrix for RF/CBC/DIFF/1/boruta model when tested on the external dataset at classification threshold of 0.3. Positive label 1 refers to a positive blood culture result. Y axis represents actual value, X axis represents predicted value.

Figure 7:


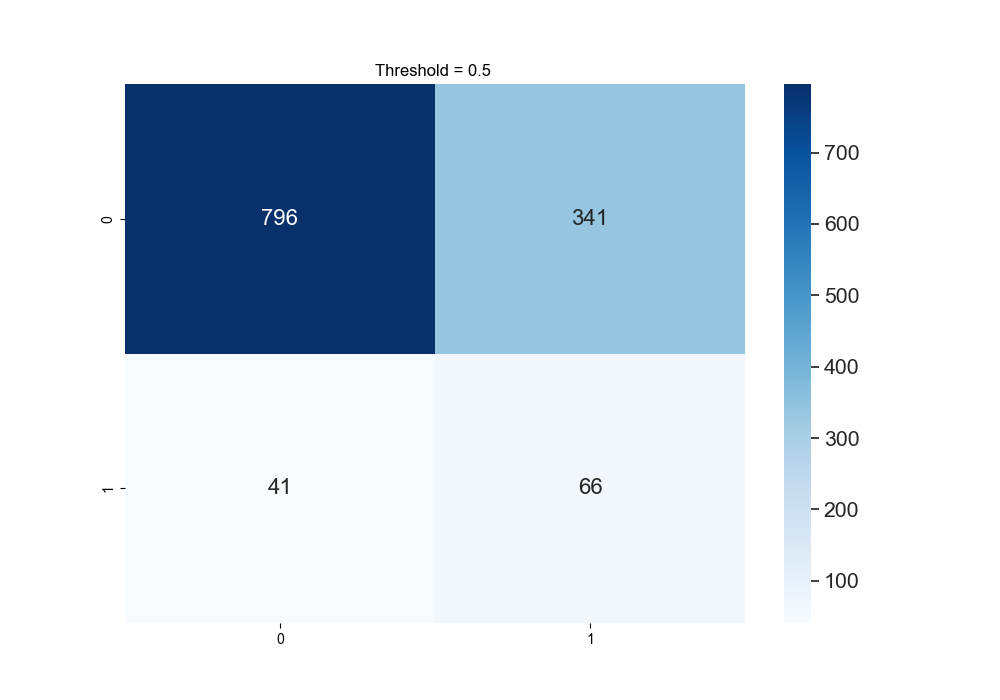


Confusion matrix for RF/CBC/DIFF/1/boruta model when tested on the external dataset at classification threshold of 0.5. Positive label 1 refers to positive blood culture result. Y axis represents actual value, X axis represents predicted value.

Figure 8:


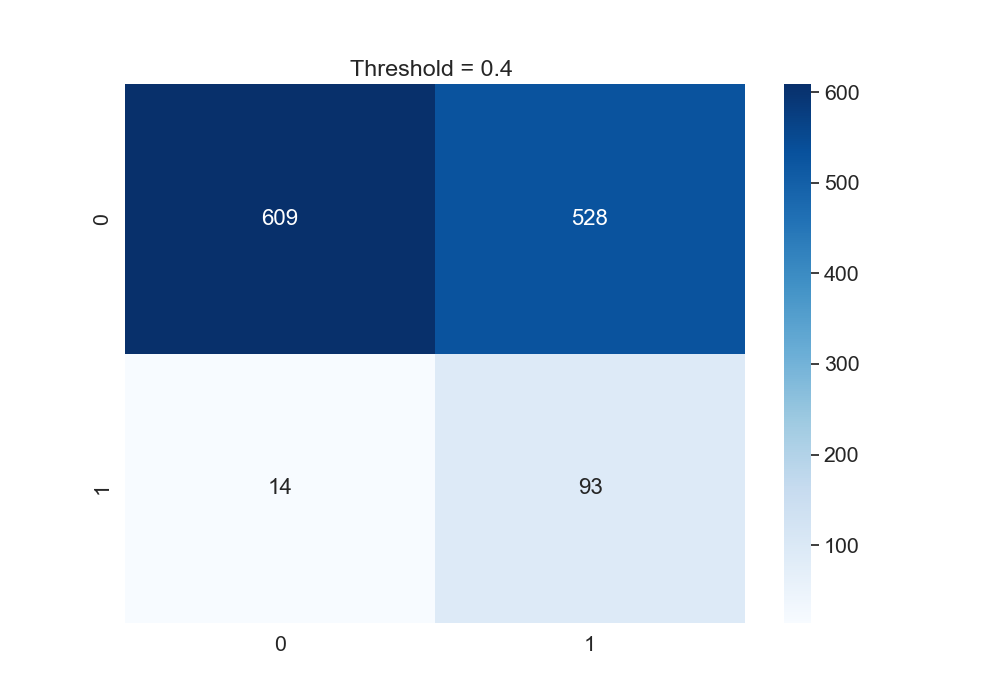


Confusion matrix for RF/CBC/DIFF/1/boruta model when tested on the external dataset at classification threshold of 0.4. Positive label 1 refers to positive blood culture result. Y axis represents actual value, X axis represents predicted value.

Figure 9:


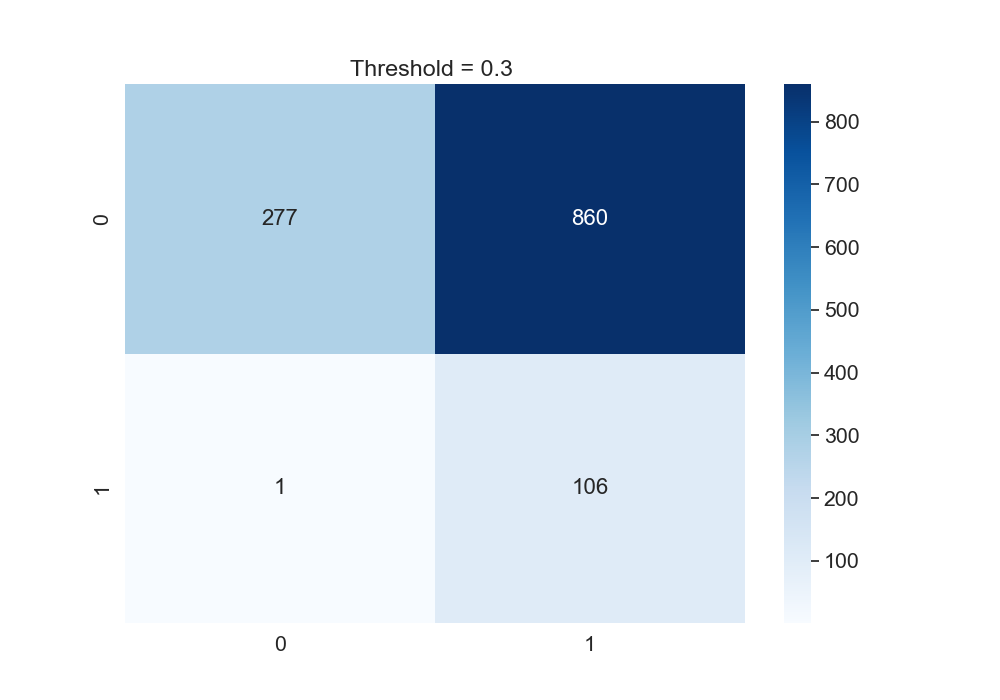


Confusion matrix for RF/CBC/DIFF/1/boruta model when tested on the external dataset at classification threshold of 0.3. Positive label 1 refers to positive blood culture result. Y axis represents actual value, X axis represents predicted value.
